# Data of NODDI diffusion metrics in the brain and computer simulation of hybrid diffusion imaging (HYDI) acquisition scheme

**DOI:** 10.1016/j.dib.2016.03.063

**Published:** 2016-03-26

**Authors:** Chandana Kodiweera, Yu-Chien Wu

**Affiliations:** a Department of Psychological and Brain Sciences, Dartmouth Brain Imaging Center, Dartmouth College, 6207, Moore Hall, Hanover, NH, 03755 USA; b Department of Radiology and Imaging Sciences, Indiana University School of Medicine, Goodman Hall, 355 West 16th Street, Suite 4100, Indianapolis, IN 46202, USA

**Keywords:** NODDI, HYDI, Diffusion, Magnetic Resonance Imaging, White matter

## Abstract

This article provides NODDI diffusion metrics in the brains of 52 healthy participants and computer simulation data to support compatibility of hybrid diffusion imaging (HYDI), “*Hybrid diffusion imaging”*[1] acquisition scheme in fitting neurite orientation dispersion and density imaging (NODDI) model, “*NODDI: practical in vivo neurite orientation dispersion and density imaging of the human brain”*[2]. HYDI is an extremely versatile diffusion magnetic resonance imaging (dMRI) technique that enables various analyzes methods using a single diffusion dataset. One of the diffusion data analysis methods is the NODDI computation, which models the brain tissue with three compartments: fast isotropic diffusion (e.g., cerebrospinal fluid), anisotropic hindered diffusion (e.g., extracellular space), and anisotropic restricted diffusion (e.g., intracellular space). The NODDI model produces microstructural metrics in the developing brain, aging brain or human brain with neurologic disorders. The first dataset provided here are the means and standard deviations of NODDI metrics in 48 white matter region-of-interest (ROI) averaging across 52 healthy participants. The second dataset provided here is the computer simulation with initial conditions guided by the first dataset as inputs and gold standard for model fitting. The computer simulation data provide a direct comparison of NODDI indices computed from the HYDI acquisition [1] to the NODDI indices computed from the originally proposed acquisition [2]. These data are related to the accompanying research article “*Age Effects and Sex Differences in Human Brain White Matter of Young to Middle-Aged Adults: A DTI, NODDI, and q-Space Study*” [3].

**Specifications Table**

**Table:** 

Subject area	*Neuroimaging*
More specific subject area	*Diffusion magnetic resonance imaging*
Type of data	*Figure, table*
How data was acquired	*Hybrid diffusion imaging (HYDI) at 3 T MRI scanner and computer simulation*
Data format	*Analyzed*
Experimental factors	*48 white matter ROIs in the human brain and synthetic diffusion signals*
Experimental features	*Diffusion microstructural metrics*
Data source location	*University of Wisconsin - Madison*
Data accessibility	*Data is with this article*

**Value of the data**

• The experimental data could serve as bench marker for studies on white matter microstructural measurements using either imaging or histologic approaches.
• This simulation data could serve as a reference for future studies that use HYDI acquisition for NODDI computation.
• This simulation approach could be applied on future studies to design acquisition schemes and investigate the schemes’ compatibility for diffusion compartment modeling.

## 1 Data

The experimental data (Table 1) include NODDI metrics (i.e., orientation dispersion index (ODI) and intercellular volume fraction (ICVF)) and other diffusion metrics for comparison such as DTI (i.e., axial diffusivity (AD), radial diffusivity (RD), mean diffusivity (MD), and factional anisotropy (FA)) and q-space analysis (i.e., zero-displacement probability (P_0_)). The means and standard deviations of diffusion metrics in 48 white matter ROIs were computed across 52 healthy participants with age between 18 and 72 years old (mean 39±14).

The HYDI acquisition scheme includes 5 shells with different diffusion-weighting *b*-value and each shell has homogeneously distributed diffusion-weighting directions [1]. Details of the HYDI diffusion-encoding scheme are shown in Table 2. We simulated different combinations of HYDI shells to compute NODDI diffusion indices. The HYDI shell combination protocols are: p12, p123, p1234, and p12345 (Table 3). The p12 protocol comprises the first and second HYDI shell with *b*-value of 375 and 1500 s/mm^2^, respectively. The p123 protocol comprises the 1st, 2nd and 3rd HYDI shell. Similarly, p1234 comprises the 1st to 4th HYDI shell and p12345 has all 5 shells. The NODDI-p14 is the diffusion-encoding scheme recommended by [2]. The NODDI diffusion model yields microstructural indices including the intracellular volume fraction (ICVF), fiber orientation dispersion index (ODI), and free water volume fraction (FISO). The estimates of these microstructural indices will be compared between the HYDI protocols and the NODDI-p14.

## 2 Experimental design, materials, and methods

The 48 white matter ROIs of the experimental data in Table 1 were defined through intersecting the white matter atlas, Johns Hopkins University (JHU) ICBM-DTI-81 [4] with the common white matter skeleton created from all of the subjects similar to [3]. The diffusion metrics of NODDI, DTI, and q-space were averaged within the ROIs and then across the 52 participants. The standard deviations reflect the variations of ROI means across the participants.

**Fig. 1:**
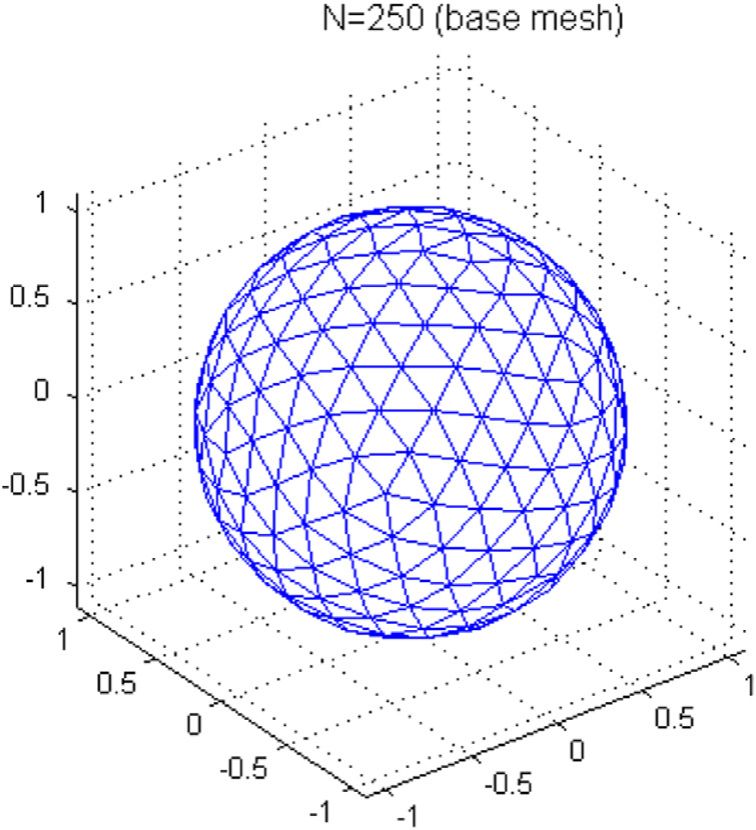
The vertices represent the fiber orientations on the unit spherical surface. There are 250 uniformly distributed vertices in this figure.

The tissue diffusion properties were simulated using SynthMeas.m provided in the NODDI Matlab toolbox (http://mig.cs.ucl.ac.uk/index.php?n=Tutorial.NODDImatlab). According to the experimental data in Table 1, we designed the ground truth and initial inputs for computer simulation: ICVF = [0.2, 0.4, 0.5, 0.8]; kappa = [0, 0.25, 1, 4, 16]; and FISO=0. Similar design can also be found in [2]. The kappa is the Watson distribution parameter that determines the estimation of ODI [5]. The intracellular axial diffusivity is set as 1.7×10^−3^ mm^2^/s, and the fast free isotropic diffusivity representing cerebrospinal fluid is set as 3×10^−3^ mm^2^/s similar to free water diffusion. There were 30 random trials at each of the 250 different fiber orientations uniformly distributed on a unit sphere (Fig. 1). Five signal-to-noise ratios (SNR) defined at *b*-value =0 s/mm^2^ was simulated: SNR_*b*0_=[20, 30, 40, 50, Infinity]. The 4 *b*_0_ SNRs yield corresponding SNRs across the 50 diffusion directions in the outermost shell, SNR_*b*=9375_=[2.22±0.48, 3.71±0.81, 4.45±0.97, 5.56±1.21], respectively. Therefore, SNR_*b*0_=20 yields the outermost-shell SNR_*b*=9375_ (2.22±0.48) that is closest to the reported measurement (i.e., 2.85±0.71) in the human brain published in [1].

**Fig. 2:**
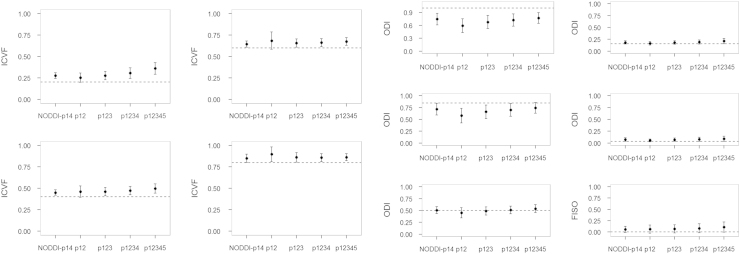
Simulated results for SNR=20. Dashed lines denote the ground truth of simulated diffusion properties (i.e., ICVF, ODI and FISO). The dot denotes the mean of the specific diffusion property estimated using NODDI-p14 and HYDI (p12, p123, p1234, and p12345) schemes. The error bar denotes the standard deviation across the 30 random trials, 250 fiber directions and the rest diffusion properties.

**Fig. 3:**
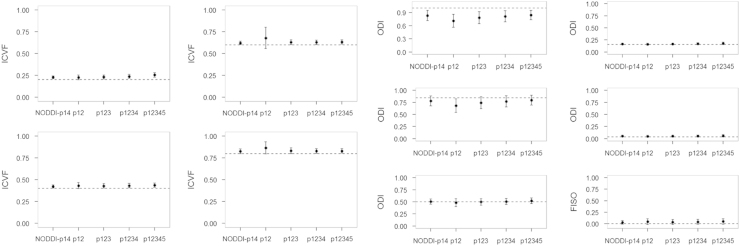
Simulated results for SNR=30. Dashed lines denote the ground truth of simulated diffusion properties (i.e., ICVF, ODI and FISO). The dot denotes the mean of the specific diffusion property estimated using NODDI-p14 and HYDI (p12, p123, p1234, and p12345) schemes. The error bar denotes the standard deviation across the 30 random trials, 250 fiber directions and the rest diffusion properties.

**Fig. 4:**
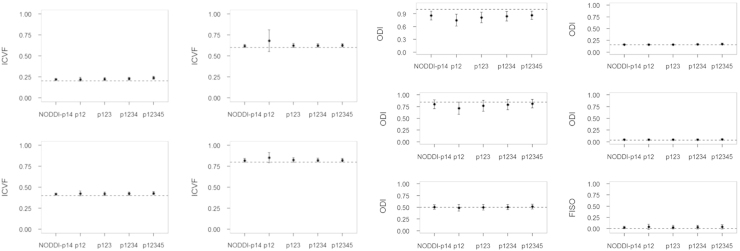
Simulated results for SNR=40. Dashed lines denote the ground truth of simulated diffusion properties (i.e., ICVF, ODI and FISO). The dot denotes the mean of the specific diffusion property estimated using NODDI-p14 and HYDI (p12, p123, p1234, and p12345) schemes. The error bar denotes the standard deviation across the 30 random trials, 250 fiber directions and the rest diffusion properties.

**Fig. 5:**
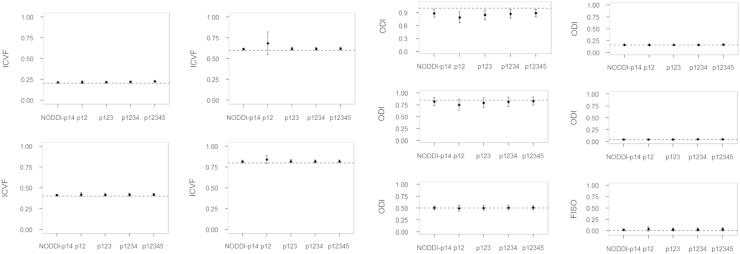
Simulated results for SNR=50. Dashed lines denote the ground truth of simulated diffusion properties (i.e., ICVF, ODI and FISO). The dot denotes the mean of the specific diffusion property estimated using NODDI-p14 and HYDI (p12, p123, p1234, and p12345) schemes. The error bar denotes the standard deviation across the 30 random trials, 250 fiber directions and the rest diffusion properties.

**Fig. 6:**
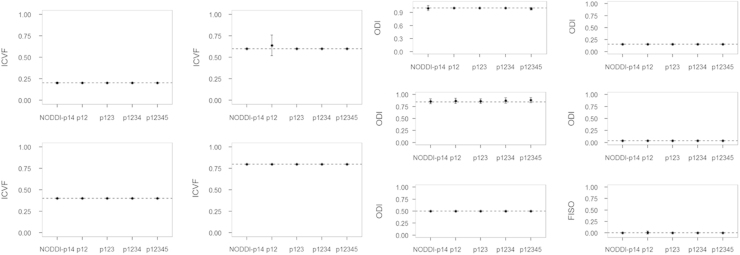
Simulated results for SNR = infinity (i.e., noise=0). Dashed lines denote the ground truth of simulated diffusion properties (i.e., ICVF, ODI and FISO). The dot denotes the mean of the specific diffusion property estimated using NODDI-p14 and HYDI (p12, p123, p1234, and p12345) schemes. The error bar denotes the standard deviation across the 30 random trials, 250 fiber directions and the rest diffusion properties.

The simulation results are shown in Fig. 2, Fig. 3, Fig. 4, Fig. 5, Fig. 6. For all of the ICVF and kappa conditions, the NODDI-p14 and HYDI p12345 schemes did not differ significantly from each other (*p*-value > 0.05). At SNR_*b*0_=20, HYDI p12345 overestimates low ICVFs more often than NODDI-p14, but both are comparable at a high ICVF (>0.5), which is a more realistic value for white matter listed in Table 1. Both schemes underestimate high ODIs, but work fine at a low ODI (<=0.5), which is again more realistic for white matter listed in Table 1. As the SNR increases, both NODDI-p14 and HYDI eventually reach the ground truth. Between the combinations of HYDI shells, p12 tends to underestimate ODI in high ODI tissue model at low SNR and also overestimate ICVF in high ICVF. Other combinations p123, p1234, and p12345 were comparable across simulated tissue properties and SNRs.

## Figures and Tables

**Table 1:** Mean and standard deviation (std) of the diffusion metrics in 48 white matter ROIs [4] across 52 subjects.

	**Da (10**^**−6**^**mm**^**2**^**/s)**		**Dr (10**^**−6**^**mm**^**2**^**/s)**		**MD (10**^**−6**^**mm**^**2**^**/s)**		**FA**		**Po**		**ODI**		**ICVF**		**FISO**	
**ROI**	**Mean**	**Std**	**Mean**	**Std**	**Mean**	**Std**	**Mean**	**Std**	**Mean**	**Std**	**Mean**	**Std**	**Mean**	**Std**	**Mean**	**Std**
ACR-L	840	40	392	4	541	36	0.470	0.033	0.463	0.042	0.292	0.023	0.655	0.057	0.149	0.033
ACR-R	848	37	395	4	546	34	0.467	0.035	0.457	0.039	0.285	0.024	0.656	0.055	0.160	0.029
ALIC-L	858	45	316	3	497	29	0.556	0.038	0.473	0.038	0.266	0.031	0.676	0.050	0.111	0.022
ALIC-R	876	51	322	3	506	32	0.556	0.037	0.461	0.038	0.260	0.026	0.682	0.053	0.124	0.025
BCC	1184	64	380	8	648	70	0.628	0.054	0.510	0.056	0.141	0.016	0.712	0.038	0.230	0.047
CGC-L	872	41	348	2	522	23	0.521	0.028	0.468	0.036	0.242	0.026	0.608	0.046	0.090	0.027
CGC-R	844	39	369	2	527	21	0.485	0.032	0.443	0.035	0.265	0.030	0.581	0.048	0.085	0.026
CGH-L	855	54	469	5	598	45	0.388	0.031	0.322	0.026	0.344	0.027	0.502	0.045	0.112	0.050
CGH-R	852	44	442	3	579	32	0.412	0.031	0.338	0.026	0.337	0.025	0.507	0.043	0.094	0.041
CP-L	1007	57	269	3	515	38	0.682	0.038	0.621	0.073	0.190	0.017	0.768	0.041	0.148	0.027
CP-R	1049	63	293	5	545	48	0.671	0.043	0.590	0.067	0.187	0.017	0.775	0.042	0.171	0.031
CST-L	883	83	332	7	516	72	0.584	0.042	0.686	0.134	0.208	0.019	0.769	0.053	0.181	0.049
CST-R	887	85	333	7	517	68	0.587	0.040	0.685	0.144	0.208	0.023	0.772	0.053	0.184	0.047
EC-L	835	45	399	5	544	44	0.455	0.034	0.379	0.033	0.320	0.026	0.531	0.037	0.075	0.037
EC-R	844	39	410	4	555	35	0.444	0.031	0.364	0.028	0.317	0.021	0.528	0.034	0.087	0.032
Fx	1716	141	830	16	1126	153	0.477	0.055	0.231	0.050	0.168	0.033	0.770	0.077	0.622	0.091
Fx-ST-L	1025	73	448	7	641	71	0.510	0.040	0.386	0.057	0.236	0.020	0.593	0.045	0.214	0.065
Fx-ST-R	1092	73	500	7	697	68	0.486	0.038	0.347	0.051	0.244	0.024	0.579	0.042	0.262	0.063
GCC	1325	93	530	8	795	83	0.575	0.033	0.411	0.045	0.180	0.018	0.722	0.047	0.336	0.054
ICP-L	817	46	340	3	499	27	0.526	0.038	0.569	0.032	0.248	0.028	0.683	0.038	0.125	0.039
ICP-R	810	46	322	3	484	27	0.539	0.039	0.590	0.037	0.247	0.030	0.683	0.038	0.105	0.032
MCP	921	90	362	6	549	66	0.569	0.045	0.662	0.107	0.220	0.028	0.809	0.050	0.204	0.045
ML-L	884	64	288	3	487	22	0.615	0.048	0.575	0.035	0.180	0.024	0.642	0.029	0.097	0.028
ML-R	900	75	287	2	491	24	0.625	0.047	0.571	0.031	0.175	0.023	0.638	0.031	0.096	0.031
PCR-L	863	79	397	6	552	65	0.494	0.032	0.530	0.049	0.224	0.016	0.628	0.053	0.117	0.046
PCR-R	847	55	378	4	534	44	0.503	0.029	0.528	0.044	0.230	0.017	0.616	0.048	0.101	0.033
PCT	767	44	328	3	474	25	0.514	0.036	0.598	0.032	0.254	0.026	0.713	0.043	0.102	0.034
PLIC-L	860	32	259	2	460	22	0.643	0.027	0.646	0.032	0.209	0.018	0.743	0.036	0.105	0.020
PLIC-R	875	35	264	2	468	23	0.640	0.027	0.629	0.027	0.205	0.016	0.736	0.033	0.111	0.021
PTR-L	1072	99	418	9	636	89	0.580	0.038	0.488	0.051	0.161	0.022	0.634	0.053	0.191	0.064
PTR-R	962	64	332	5	542	48	0.608	0.036	0.548	0.063	0.180	0.022	0.656	0.057	0.132	0.035
RLIC-L	904	54	318	4	513	37	0.600	0.031	0.556	0.040	0.199	0.021	0.686	0.038	0.129	0.032
RLIC-R	905	44	328	3	520	29	0.588	0.030	0.540	0.037	0.211	0.019	0.681	0.040	0.135	0.031
SCC	1102	42	236	3	525	30	0.749	0.027	0.666	0.042	0.122	0.013	0.764	0.038	0.125	0.027
SCP-L	1316	106	534	8	795	82	0.579	0.033	0.454	0.036	0.154	0.026	0.724	0.041	0.329	0.057
SCP-R	1243	91	457	5	719	58	0.607	0.031	0.490	0.029	0.145	0.024	0.718	0.039	0.295	0.048
SCR-L	760	35	337	3	478	30	0.501	0.030	0.595	0.039	0.268	0.016	0.689	0.044	0.103	0.024
SCR-R	760	29	344	2	483	22	0.488	0.025	0.586	0.033	0.273	0.018	0.692	0.041	0.114	0.026
SFO-L	812	73	361	6	511	64	0.500	0.038	0.499	0.057	0.286	0.029	0.661	0.061	0.117	0.047
SFO-R	810	49	361	4	510	35	0.491	0.037	0.483	0.045	0.282	0.032	0.645	0.069	0.112	0.038
SLF-L	745	30	332	2	470	23	0.494	0.025	0.605	0.036	0.269	0.018	0.699	0.040	0.096	0.021
SLF-R	763	29	344	2	484	20	0.489	0.023	0.571	0.035	0.275	0.020	0.693	0.042	0.107	0.025
SS-L	967	73	419	5	602	52	0.511	0.029	0.427	0.037	0.225	0.026	0.594	0.041	0.172	0.047
SS-R	951	57	387	3	575	36	0.536	0.028	0.447	0.039	0.237	0.028	0.635	0.049	0.167	0.032
TAP-L	2053	266	1328	25	1569	251	0.329	0.048	0.157	0.055	0.179	0.042	0.819	0.079	0.758	0.118
TAP-R	1788	190	1026	17	1280	176	0.414	0.054	0.230	0.064	0.143	0.028	0.759	0.089	0.624	0.123
UNC-L	864	66	438	6	580	56	0.419	0.048	0.329	0.037	0.306	0.052	0.455	0.033	0.061	0.047
UNC-R	926	76	464	8	618	72	0.425	0.055	0.303	0.045	0.296	0.053	0.474	0.054	0.116	0.103

**Table 2:** HYDI shells, number of diffusion encoding directions (Ne), and the corrosponding *b*-values.

HYDI shell	Ne	*b-*value (s/mm^2^)
	1	0
1st	6	375
2nd	21	1500
3rd	24	3375
4th	24	6000
5th	50	9375
total	126	

**Table 3:** Diffusion encoding protocols

**Protocol**	***b*-value(s/mm**^**2**^**), (number of diffusion directions)**
NODDI-p14	*b*=711(30), *b*=2855(60)
p12	*b*=375(6), *b*=1500(21)
p123	*b*=375(6), *b*=1500(21), *b*=3375(24)
p1234	*b*=375(6), *b*=1500(21), *b*=3375(24), *b*=6000(24)
p12345	*b*=375(6), *b*=1500(21), *b*=3375(24), *b*=6000(24), *b*=9375(50)
